# Job Satisfaction in the Shrimp Trawl Fisheries of Chennai, India

**DOI:** 10.1007/s11205-012-0055-3

**Published:** 2012-05-05

**Authors:** Maarten Bavinck

**Affiliations:** Nieuwe Prinsengracht 130, 1018 VZ Amsterdam, The Netherlands

**Keywords:** Job satisfaction, Trawl fishing, Tamil Nadu, India, Blue revolution

## Abstract

Shrimp trawling represents an important fishing métier in South India, generating high levels of employment and economic value. It is also a contested métier, ostensibly contributing to environmental degradation and social inequality. This paper investigates the job satisfaction of crew members (captains and workers) on board the shrimp trawlers of Chennai (former Madras). Research took place in 2007 and 2008 (N = 137). Results suggest a general satisfaction with being in the fishery. However, a little over three-fifths of fishers said they would be willing to change fishing métier and about one-half said they would leave the occupation. About one-half also said they would not advise a young person to enter the occupation. The tendency to move away from the fishery is argued to reflect a growing pessimism about the future of the shrimp trawl fisheries, but also an increasing awareness of other economic opportunities.

## Introduction

The research on which this paper is based is part of an interdisciplinary effort to compare and evaluate the costs and benefits of capture fisheries (see introductory paper to this special issue). The social scientists involved focus on perhaps the most human of values: the extent to which various fisheries generate wellbeing for those who participate (Smith and Clay 2010; Bavinck and Monnereau 2007). To this end, they made use of what is called the job satisfaction approach. The study of job satisfaction originates in the field of labour studies, and has been used to understand and improve the working conditions of employees in various sectors of industrial economies. Starting in the late 1970s, some scholars have adopted this methodology to understand job satisfaction of fishers in different geographic locations and fishing *métiers*. Most of these studies deal with fisheries in North America (Binkley 1995).

The challenge faced by the group was to expand the study of job satisfaction to other regions and to develop a framework which would allow for inter-cultural comparison. The present paper reports on one of these test exercises carried out among shrimp trawl fishers in the fishing harbour of Chennai in southeast India. It must be remembered that capture fishing in India is still largely a caste-based occupation and that members of these castes generally reside at the bottom of the caste hierarchy. A modernisation process initiated by the government has recently brought more prosperity and changed public perceptions of the profession. Section 2 introduces the fisheries of Chennai in the context of the region and describes the *métier* selected for study. The following sections present the methodology used and the results of the survey.

## The Fisheries of Chennai

The mega-city of Chennai (6.6 million inhabitants) is the capital of the state of Tamil Nadu. Although it boasts several fishing neighbourhoods, most of its fishing industry is concentrated in the fishing harbour of Royapuram, in the north of the city. Royapuram is a boisterous and rough area, largely inhabited by people working in the fishing sector (Bavinck 2001). The fishing harbour, which was completed in 1984, offers berthing space to approximately 700 so-called mechanised boats, most of which are used for inshore trawling. A substantial number of small-scale fishers also make use of the harbour. Many of them originate from villages to the north of Chennai, having shifted their operations to this location following large-scale erosion of their landing beaches. These fishers make use of capital-extensive fibreglass boats or wooden *kattumarams* that are propelled by oar, sail, or outboard engine.

The construction and operation of the Chennai fishing harbour is closely connected to the Blue Revolution in India, which fundamentally reshaped the fisheries of Tamil Nadu in the decades after Independence in 1947, and created new tensions that revolved around issues of social justice and environmental sustainability (Johnson and Bavinck 2010; Subramanian 2009). In the decades that followed Independence, the government of India modernised the extensive, although in popular perception backward, fisheries sector. Rather than building on existing technologies and insights, it borrowed heavily from Western experience. The mechanised boat sub-sector, as it is known locally, depended on the realisation of modern infrastructure, such as harbours, preservation and transport facilities, and boat building yards. The Chennai fishing harbour is one of the nodes of the new, mechanised boat fishery.

**Fig. 1:**
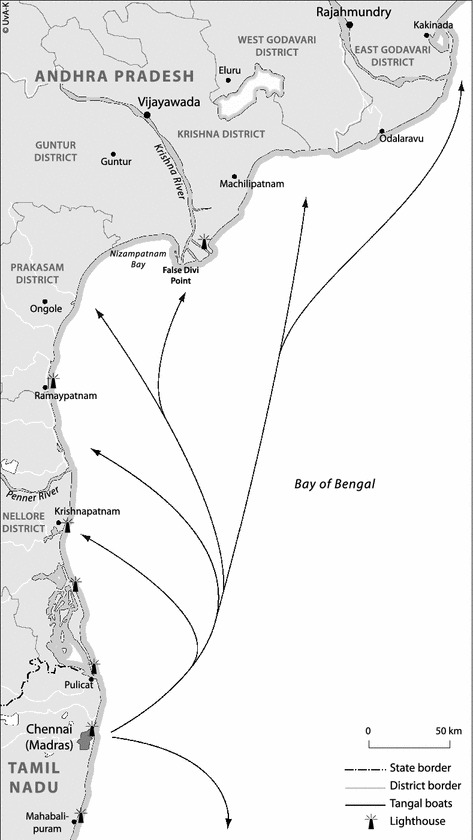
Fishing grounds of chennai shirmp trawl fishers (*source* UvA Kaartenmakers)

The history of the mechanised boat fishery in Tamil Nadu has been recounted in detail by numerous authors (*c.f*. Bavinck 2001; Ram 1991; Subramanian 2009). A few points are, however, noteworthy. The first is that the emergence of the mechanised boat fishery in India has largely been synonymous with trawling, mainly for shrimp. Secondly, mechanised boat fishers have tended to focus on inshore fishing grounds, mainly because of their fecundity. When the fishing grounds in the vicinity of Chennai became exhausted, attention shifted northwards to the inshore waters of Andhra Pradesh (see Fig.1). As Andhra boasts many rich fishing grounds, which are relatively underexploited, the mechanised boat fishers of Chennai started making longer forays northward, up to a distance of 600 km, and adapted their craft for this purpose. Whereas the first mechanised boats in Chennai were less than 32 ft in length, current craft measure up to 50 ft and engine power has increased from 10 to 110 hp. Holds have increased in size and many crews now make use of GPS, sonar and telecommunication equipment. Fishing trips can last up to 21 days, with a large portion of that time used for travel to and from the fishing grounds.

The dependence of the Chennai fleet on stocks in northern waters has, however, brought trawl fishers into regular conflict with small-scale fishers in Andhra Pradesh, the result of which is a protracted ‘fishing war’ (Bavinck 2011). The management of such tensions is an important issue in the Chennai fisheries.

Since its inception, some differentiation has occurred in the mechanised boat fishery. Although most crews (an estimated 500) still carry out trawling, a growing number of owners—the Fisheries Department registered 173 of such units in 2008—have converted their craft for gillnet and long line fishing. This is a result of the declining price of shrimp on the world market and the perceived availability of high-value pelagic fish in offshore waters. In addition, there is a difference between richer boat owners and those at the lower end of the socioeconomic status hierarchy. Whereas richer boat owners often possess several craft capable of long-distance fishing, their poorer colleagues generally make do with a single, small craft (known as ‘day boats’). Moreover, because the boats belonging to poorer owners are often old and in need of frequent repair, they are not popular among potential crew members, who prefer to serve on large and better equipped boats. This research has focused on the more profitable long-distance shrimp trawl fishery of Chennai, leaving the small-scale fishery, the so-called day boat fishery, and other varieties of mechanised boat operations aside.

Compared with small-scale fishing operations, the mechanised boat fishery of Chennai is capitalist in nature. Ownership of equipment is concentrated in a few hands and boat owners generally do not go out fishing themselves. The captain—locally known as the ‘driver’—is in charge of daily operations and responsible for recruiting a crew of up to eight persons. Crew members do shifts in order to minimise the time spent in the harbour and receive a share of the total proceeds. Boat owners rely heavily on merchant loans and experience heavy pressure to keep their craft in continuous operation.

Higher oil prices and declining prices of seafood have plunged the mechanised boat fisheries of Tamil Nadu into a deep and prolonged crisis. Although Chennai has experienced difficult times, particularly in the first years of the new millennium, the crisis now seems to have diminished. One of the indications is that boat building has recommenced after years of recession. At the same time, mechanised boat fishers generally agree that a severe resource crisis is affecting the fisheries. This is why they have also agreed to a 45-day closed season, first announced by the government in 2000 and implemented in April and May of every year thereafter (Bavinck et al. 2008).

With all these fisheries available, the Chennai fishing harbour is an extremely lively place. In addition to fishers, many people are employed in service industries, processing units, trade enterprises and transportation. With people moving in and out of the harbour area, and in and out of the fisheries as well, the fishing sector is particularly hard to oversee and regulate. This is all the more true because fisher organisations dispute the government’s right to intervene in their affairs. Five professional organisations of boat owners control the mechanised boat fishery. A union of fisher neighbourhood organisations called *Aikya Panchayat Sabai* is in charge of the harbour area and the small-scale fisheries. The Fisheries Department and the Port Trust represent various government interests and negotiate with fisher organisations as to the implementation of plans. To top this off, the Minister of Fisheries, who is frequently elected from this area, personally involves himself in harbour affairs. Management of the fisheries is therefore highly complicated and opaque.

## Research Methodology

The job satisfaction research on which this paper is based was carried out at two points of time, with an interval of 1 year. It coincided with the implementation of an undergraduate course on marine resource management at Anna University, Chennai in February 2007 and February 2008. The Dutch and Indian students participating in the course were divided by the researcher into survey teams of two to four persons and spent several days conducting the survey.

On Days 1 and 2, the students were taken to the harbour area and given assignments intended to familiarise them with the location and fishing activities taking place there. The students spent Day 1 drawing maps of different parts of the fishing harbour and Day 2 exploring aspects of the trawl fishing *métier*.

Day 3 was spent largely on interview training. The researcher went through and explained the interview schedules—the standardised English version as well as a Tamil translation, highlighting possible points of confusion and answering questions.^1^ The researcher subsequently organised role-plays with students playing the parts of interviewers and respondents. These were discussed afterwards.

The survey teams (7 in 2007 and only 2 in 2008) spent the mornings of Days 4–6 in the harbour area, identifying respondents and conducting interviews. Respondents were selected using snowball sampling, with each survey team commencing work in a different part of the harbour area. The researcher stayed in the vicinity and attended a number of interview sessions. Questions or problems were discussed in the evenings. The inability of having a private conversation was one of the recurring problems. In many cases, the interviews attracted bystanders who involved themselves in the discussion. The students spent Day 7 analysing the results of their efforts, which were subsequently presented to and discussed with an external academic audience on Day 8.

## Analysis

### Research Instrument and Sample

The 2007 research group administered 90 surveys and the 2008 group 47 surveys in Royapuram fishing harbour. The difference in the number of surveys conducted was a result of the smaller number of students participating in 2008. The procedure was the same in both years: the survey teams spread out through the harbour area in the morning hours and conducted interviews with trawl fishers on the dock as they took a break from loading, unloading and cleaning the boats. In many cases, other fishers congregated around. The interview period coincided with a relatively low fishing season, which may have negatively influenced opinions on job satisfaction.

### Sample Characteristics

Table 1 indicates that the average age of fishers interviewed is almost 36 and the education level just under 6 years. The average household size is 5.4 and interviewees have about 18 years of fishing experience. Most respondents (107) are married, while the remainder (28) are single. The sample can therefore be characterised as consisting of married men with a family to take care of, with a lesser representation of unmarried men.

**Table 1:** Distribution of demographic variables

	N	Min.	Max.	Mean	SD
Age	137	17	65	35.68	10.228
Education	137	0	15	5.80	3.902
Years fishing	136	0	50	18.41	10.465
Household size	136	0	66	5.41	5.773

### Job Satisfaction Items and Scales

The central tendencies for job satisfaction items are in Table 2. Most scores are above the mid-point of 3, indicating general satisfaction with being in the fishery. Respondents expressed greatest satisfaction for the items cleanliness and healthfulness of the occupation, the ability to be one’s own boss, the community in which they live, and time for recreation. Lowest scores were recorded for predictability and level of earnings, the performance of government officials, the condition of the landing place, and the condition of the fish stocks.

**Table 2:** Distribution of scores on job satisfaction items

	N	Min.	Max.	Mean	SD
Safety	137	1	11	3.24	1.593
*Predictability of earnings*	*137*	*1*	*5*	*2.85*	*0.984*
*Earnings*	*137*	*1*	*5*	*3.06*	*0.906*
Mental pressure	137	1	5	3.27	0.912
**Cleanliness**	**137**	**1**	**5**	**3.74**	**1.249**
Hours fishing	137	1	5	3.32	1.064
**Healthfulness**	**137**	**1**	**5**	**3.73**	**0.818**
Fatigue	136	1	5	3.57	0.747
Time to fishing grounds	137	1	5	3.38	0.815
Food security	137	1	5	3.42	0.945
Catch level	136	1	5	3.56	1.140
Time at sea	137	1	5	3.71	1.079
Time away from home	137	1	5	3.15	0.946
**Being your own boss**	**136**	**2**	**5**	**3.93**	**1.020**
**Community in which you live**	**137**	**1**	**5**	**3.96**	**0.919**
**Time for recreation with family**	**136**	**1**	**5**	**3.73**	**0.970**
Challenge of job	136	1	5	3.53	0.902
Adventure of job	136	1	5	3.33	0.903
Doing something worthwhile	136	1	5	3.62	0.935
Conflict in fishery	136	1	5	3.18	1.010
Conflict resolution	136	1	5	3.51	0.816
Overall management	136	1	5	3.41	0.839
*Performance of Gov. Off.*	*136*	*1*	*5*	*2.74*	*1.104*
Rules and regulations	136	1	5	3.29	1.088
Influence over management	136	1	5	3.35	0.955
*Condition of landing place*	*136*	*1*	*5*	*3.04*	*1.017*
*Condition of fish stocks*	*136*	*1*	*5*	*2.66*	*0.983*

Bold = 5 highest, italics = 5 lowest

The choice of lowest scoring items is confirmed by qualitative information gathered in this fishery. The decline of catches and shrimp prices and the increase of running costs have tended to reduce earnings in the shrimp fishery in the past decade. Dissatisfaction about the landing site is related to its congestion and lack of cleanliness. Finally, there is widespread frustration with the perceived partiality and corruption of officials of the Fisheries Department and their perceived inability to address the serious problems affecting the fishing sector.

The highest scoring items can also be explained by the context. Once at sea, fishers the world over appreciate the ability to be their own boss and the healthfulness of the occupation (Binkley 1995; Pollnac and Poggie 2008). Satisfaction with the community probably links up to the traditional, caste-based nature of the occupation and the fact that fishers tend to congregate in the same neighbourhoods. High satisfaction expressed with the time for recreation is more surprising, in view of the fact that fishers are out at sea for many days at a time and only return to shore for a brief interlude. It may, however, be caused by the timing of the survey, which took place during the slow fishing season when there is more time ashore and less at sea.

**Fig. 2:**
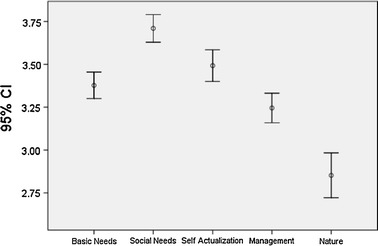
Mean values and confidence intervals for job satisfaction categories

Figure 2 presents the mean scores per job satisfaction category. Whereas scores for satisfaction with Basic Needs, Social Needs, and Self-Actualisation are above the mid-point of 3, scores for Management hover around the mid-point, and scores for Nature are significantly below this level. Other evidence from the fishery confirms the supposition that concerns about the state of fish stocks and the quality of management weigh heavily on fishers’ minds.

As a first step in the analysis we look at the relationships between basic background social data and the job satisfaction categories (Table 3). As can be seen in Table 3, the level of satisfaction on Basic Needs decreases as household size increases. This correlates with the general dissatisfaction about the level and predictability of earnings. Finally, satisfaction with Social Needs increases as educational level decreases and years fishing experience increases.

**Table 3:** Correlations between job satisfaction categories and selected social variables

	Basic needs	Social needs	Self actualise	Manage	Nature
Age	0.039	0.168	−0.090	0.068	−0.087
Education	−0.060	−0.246*	0.146	0.122	−0.037
Years fishing	−0.063	0.212*	−0.058	0.097	−0.089
Household size	−0.171*	0.018	0.044	−0.059	−0.110

* *p* < 0.05

Tables 4 and 5 examine mean values on job satisfaction categories across marital and crew status. Table 4 indicates that married fishers are more satisfied with regard to Social Needs and less satisfied concerning Self-Actualisation. Turning to Table 5, we can see that captains differ marginally from crew members. The only statistically significant difference is with regard to Social Needs, on which captains score slightly higher.

**Table 4:** Job satisfaction category mean values by marital status

	Marital status	N	Mean	SD	*t* value
Basic needs	Single	28	3.35714	0.450098	0.268
	Married	107	3.38318	0.459667	
Social needs	Single	28	3.54286	0.552052	2.126*
	Married	107	3.75327	0.441763	
Self-actualisation	Single	28	3.79762	0.398962	4.173*
	Married	108	3.41358	0.548519	
Management	Single	28	3.29762	0.499706	0.613
	Married	108	3.23148	0.511464	
Nature	Single	28	2.94643	0.749559	0.717
	Married	108	2.82870	0.779893	

Bold = equal variance not assumed

* *p* < 0.05

**Table 5:** Job satisfaction category mean values by crew position

	N	Mean	SD	*t* value
Basic needs				
Captain	56	3.377	0.500	0.025
Crew	79	3.379	0.426	
Social needs				
Captain	54	3.863	0.469	3.183*
Crew	81	3.607	0.449	
Self-actualisation				
Captain	55	3.497	0.584	0.076
Crew	81	3.490	0.517	
Management				
Captain	55	3.221	0.496	0.451
Crew	81	3.261	0.518	
Nature				
Captain	55	2.873	0.835	0.245
Crew	81	2.840	0.732	

* *p* < 0.05

Previous studies of job satisfaction did not include the items in the Management and Nature categories. Hence, we thought it would be worthwhile to examine relationships between these two categories and the other three categories that have traditionally been included in job satisfaction research. The results of this analysis are found in Table 6. Table 6 indicates that scores on the Nature category increase as scores on Social Needs increase.

**Table 6:** Correlations between job satisfaction categories and attitudes towards management and nature

	Management	Nature
Basic needs	−0.106	0.104
Social needs	0.055	0.217*
Self-actualisation	0.099	0.071

* *p* < 0.05

Table 7 shows a breakdown of responses per job satisfaction category per survey year (2007 and 2008). This table indicates that average responses vary somewhat by time period, perhaps reflecting different conditions in the fishery or the performance of the teams involved in research. In both time periods, however, satisfaction with the meeting of Social Needs obtained the highest scores, and Nature the lowest.

**Table 7:** Job satisfaction category mean values by year

	Year	N	Mean	SD	*t* value
Basic needs	Year 1	90	3.47071	0.420387	3.484*
	Year 2	45	3.19192	0.472466	
Social needs	Year 1	90		0.506714	1.660
	Year 2	45	3.80444	0.382549	
Self-actualisation	Year 1	91	3.57875	0.570543	2.691*
	Year 2	45	3.31852	0.437830	
Management	Year 1	91	3.16117	0.547413	2.809*
	Year 2	45	3.41481	0.366911	
Nature	Year 1	91	2.76374	0.786485	1.934
	Year 2	45	3.03333	0.718268	

* *p* < 0.05

### Analysis of Willingness to Change

This section of the paper examines factors influencing willingness to change fishing métier, leave the occupation of fishing or advise a young person to fish. A little over three-fifths of fishers said they would be willing to change fishing métier and about one-half said they would leave the occupation (Tables 8, 9, 10). About one-half also said they would not advise a young person to enter the occupation.

**Table 8:** Per cent distribution of willingness to change fishing métier by year

	Year 1	Year 2	Total	N
Yes	60.440	70.455	63.704	86.000
No	39.560	29.545	36.296	49.000
N	91.000	44.000		135.000

χ^2^ = 1.287; *df* = 1; *p* > 0.05

**Table 9:** Per cent distribution of willingness to change occupation by year

	Year 1	Year 2	Total	N
Yes	48.235	63.636	53.488	69.000
No	51.765	36.364	46.512	60.000
N	85.000	44.000		129.000

χ^2^ = 2.764; *df* = 1; *p* > 0.05

**Table 10:** Percent distribution of willingness to advise young to fish by year

	Year 1	Year 2	Total	N
Yes	46.591	61.364	51.515	68.000
No	53.409	38.636	48.485	64.000
N	88.000	44.000		132.000

χ^2^ = 2.563; *df* = 1; *p* > 0.05

The tendency to move away from the fishery reflects a growing pessimism about the future of the fisheries in Royapuram, but also an increasing awareness of other economic opportunities. The latter correlates with the gradual breakdown of caste barriers in matters of occupation (Fuller 2003), and local knowledge of the many fishers who have actually moved into other economic fields.

Tables 8, 9, 10 also present a breakdown of figures per survey year (2007 and 2008). These reflect differences over time in willingness to change fishing métier and occupation, and to advise a young person to fish. Respondents in Year 2 are considerably more negative about the occupation than those in Year 1—a result the researcher is not yet able to explain but which may relate to variable socioeconomic conditions.

Turning to an examination of these responses in relation to background social variables (Tables 11, 12, 13), Table 11 indicates that fishers with more education are less favourable towards changing fishing métier. There are no statistically significant relationships in Tables 12 and 13, suggesting that neither willingness to leave the occupation of fishing and advise a young person to enter the occupation are related to any of the four variables.

**Table 11:** Mean values of social background variables by willingness to change fishing métier

	Change métier	N	Mean	SD	*t* value
Age	Yes	86	36.52	10.407	1.056
	No	49	34.59	9.878	
Education	Yes	86	5.20	3.958	2.121*
	No	49	6.65	3.603	
Years fishing	Yes	85	19.60	10.223	1.637
	No	49	16.54	10.782	
Household size	Yes	85	4.78	2.259	**1.343**
	No	49	6.55	9.090	

Bold = equal variance not assumed

* *p* < 0.05

**Table 12:** Mean values of social background variables by willingness to change occupation

	Change occupation	N	Mean	SD	*t* value
Age	Yes	69	36.59	10.561	0.837
	No	60	35.07	10.077	
Education	Yes	69	6.19	3.817	1.691
	No	60	5.05	3.811	
Years fishing	Yes	69	17.73	10.932	0.921
	No	59	19.45	10.049	
Household size	Yes	68	4.94	2.368	0.788
	No	60	5.75	8.079	

* *p* < 0.05

**Table 13:** Mean values of social background variables by willingness to advise a young person to fish

	Advise young to fish	N	Mean	SD	*t* value
Age	Yes	68	35.71	9.320	0.183
	No	64	35.38	11.414	
Education	Yes	68	6.10	3.875	0.843
	No	64	5.53	3.912	
Years fishing	Yes	68	16.77	10.133	1.512
	No	63	19.53	10.780	
Household size	Yes	67	4.79	1.665	0.916
	No	64	5.19	3.106	

* *p* < 0.05

Next, we turn to the relationships between willingness to change fishing métier, leave the occupation of fishing and advise a young person to fish and two other social variables: marital status and crew status. None of the obvious comparisons show any statistical significance, not single versus married or captain versus driver versus crew, in relation to any of the three willingness to change variables. Forty-eight per cent of the single fishers versus 68 % of married fishers say they would change fishing métier—a difference that is not statistically significant (χ^2^ = 3.532, *df* = 1 *p* > 0.05). Sixty per cent of the captains versus 66 % of the crew report they would change fishing métier—another difference that is not statistically significant (χ^2^ = 0.551, *df* = 1, *p* > 0.05). Marital status (50 % of the single versus 54 % of the married, χ^2^ = 0.159, *df* = 1 *p* > 0.05) and crew status (54 % crew, 53 % captains, χ^2^ = 0.016, *df* = 1, *p* > 0.05) are not statistically significantly related to willingness to leave the occupation of fishing. Finally, neither marital status (43 % of the single vs. 54 % of the married, χ^2^ = 1.067, *df* = 1 *p* > 0.05) nor crew status (51 % crew, 53 % captains, χ^2^ = 0.061, *df* = 1, *p* > 0.05) are statistically significantly related to willingness to advise a young person to become a fisher. An explanation for this lack of social differentiation did not emerge from the study.

Willingness to change is expected to be related to levels of job satisfaction—the higher the satisfaction, the less willing a fisher should be to change fishing métier or leave the occupation of fishing, and the more willing they should be to advise a young person to fish. Mean values on job satisfaction categories in relation to responses to these questions are examined in Tables 14 through 16. Since we predict the direction of the relationship, one-tailed statistical tests of significance are used.

**Table 14:** Mean value of job satisfaction categories by willingness to change fishing métier

	Change métier	N	Mean	SD	*t* value
Basic needs	Yes	84	3.40043	0.429917	0.673
	No	49	3.34508	0.501369	
Social needs	Yes	86	3.75814	0.453085	1.617
	No	48	3.62083	0.502317	
Self-actualisation	Yes	86	3.39535	0.532626	2.714*
	No	49	3.65306	0.526867	
Management	Yes	86	3.20349	0.538942	1.202
	No	49	3.31293	0.449579	
Nature	Yes	86	2.73837	0.738547	2.217*
	No	49	3.04082	0.802579	

* *p* < 0.05, 1-tailed test

Table 14 indicates that fishers not willing to change fishing métier score higher on the Self-Actualisation and Nature job satisfaction categories. The latter result is to be expected; the more optimistic shrimp fishers are about their fishing grounds, the less likely they are to want to shift fishing métier. The former is surprising, however, as there is no evidence that shrimp trawling offers more opportunities for self-actualisation than other fishing métiers. Fishers willing to leave the occupation of fishing score lower on the Self-Actualisation and higher (opposite of the predicted direction) on the Management categories (Table 15). The first result is logical in view of the opportunities fishing provides for self-actualisation; persons unappreciative of this are more likely to leave. Finally, the fishers who would advise a young person to enter the occupation of fishing score lower (opposite of the predicted direction) on the Self-Actualisation category (Table 16). This result also begs further study.

**Table 15:** Mean value of job satisfaction categories by willingness to change occupation

	Change occupation	N	Mean	SD	*t* value
Basic needs	Yes	68	3.40241	0.442672	0.380
	No	59	3.37134	0.479053	
Social needs	Yes	69	3.75072	0.409312	**1.230**
	No	60	3.64667	0.532810	
Self-actualisation	Yes	69	3.37681	0.539041	2.250*
	No	60	3.58333	0.497167	
Management	Yes	69	3.32367	0.494978	1.688*
	No	60	3.17222	0.523431	
Nature	Yes	69	2.83333	0.793664	0.363
	No	60	2.88333	0.766716	

Bold = equal variance not assumed

* *p* < 0.05, 1-tailed test

**Table 16:** Mean value of job satisfaction categories by willingness to advise a young person to enter the occupation of fishing

	Advise young to fish	N	Mean	SD	*t* value
Basic needs	Yes	67	3.41655	0.466818	0.694
	No	63	3.36075	0.448335	
Social needs	Yes	68	3.69412	0.408083	**0.089**
	No	63	3.70159	0.537465	
Self-actualisation	Yes	68	3.34804	0.538513	3.132*
	No	64	3.63021	0.493761	
Management	Yes	68	3.21569	0.561373	**0.772**
	No	64	3.28385	0.450044	
Nature	Yes	68	2.88971	0.727117	0.453
	No	64	2.82812	0.831993	

Bold = equal variance not assumed

* = *p* < 0.05, 1-tailed test

## Conclusions

This paper reports on the results of a job satisfaction survey among coastal shrimp trawl fishers in Chennai, India. This fishery spearheaded the Blue Revolution that was initiated in the decades after independence and can be taken as a flagship *métier* for the state of the modern, in contrast to the small-scale, fisheries.

‘Willingness to change’ is perhaps the most noteworthy topic of the survey, linking up to the widespread need among fisheries scientists and policymakers to investigate and promote alternative employment among fishers (Pauly 2006; Pollnac et al. 2001). The survey results confirm evidence that the shrimp trawl fishery in South India has, in the past decade, run into severe trouble—catches and landing prices have declined, and running costs have increased. Crew members on shrimp trawlers express dissatisfaction with the state of the stocks and the performance of management.

As a result of the situation of the shrimp trawl fishery, a large majority of respondents express a willingness to change fishing *métier*. A smaller majority indicate a willingness to move out of the fishing profession altogether, while almost half of the respondents would advise against a young person entering fishing. These results suggest that, if alternative employment would be available and other conditions are appropriate, a significant number of trawl fishers might leave the sector. A downsizing of the trawl fleet in Tamil Nadu, as investigated by Sathyapalan et al. (2008), might then be a feasible option. These results also indicate that for those involved in the fishing sector, caste identity no longer precludes a change in occupation.

Weighing against this conclusion is the fact that most respondents express satisfaction with many of the working conditions in their fishery. It is therefore not unlikely that if management succeeds in addressing the problems that affect the fishery and its waning economic performance, crew members will adjust their opinions. Moreover, whether fishers actually take the step of moving out of the profession depends in large part on the alternatives available. In the context of South India, these are likely to remain scarce for some decades to come.

## Footnotes

1 The Tamil version of the survey was later translated back into English by someone who had not participated in the research and did not know the original. This enabled the researcher to ascertain the extent to which the Tamil version matched the English version.
